# External validation of two prediction tools for patients at risk for recurrent *Clostridioides difficile* infection

**DOI:** 10.1177/1756284820977385

**Published:** 2021-01-09

**Authors:** Tessel M. van Rossen, Laura J. van Dijk, Martijn W. Heymans, Olaf M. Dekkers, Christina M. J. E. Vandenbroucke-Grauls, Yvette H. van Beurden

**Affiliations:** Amsterdam UMC, Vrije Universiteit Amsterdam, Medical Microbiology and Infection Control, Amsterdam Infection and Immunity Institute, Amsterdam UMC location VUmc, PK 2X132, De Boelelaan 1117, Amsterdam, 1081 HV, The Netherlands; Amsterdam UMC, Vrije Universiteit Amsterdam, Gastroenterology and Hepatology, Amsterdam Gastroenterology Endocrinology Metabolism Institute, Amsterdam, The Netherlands; Amsterdam UMC, Vrije Universiteit Amsterdam, Epidemiology and Data Science, Amsterdam Public Health Research Institute, Amsterdam, The Netherlands; Leiden University Medical Center, Clinical Epidemiology, Leiden, The Netherlands; Amsterdam UMC, Vrije Universiteit Amsterdam, Medical Microbiology and Infection Control, Amsterdam Infection and Immunity Institute, Amsterdam, The Netherlands; Amsterdam UMC, Vrije Universiteit Amsterdam, Gastroenterology and Hepatology, Amsterdam Gastroenterology Endocrinology Metabolism Institute, Amsterdam, The Netherlands

**Keywords:** *Clostridioides difficile*, *Clostridium difficile*, prediction models, risk factors, prognostic factors, recurrence

## Abstract

**Background:**

One in four patients with primary *Clostridioides difficile* infection (CDI) develops recurrent CDI (rCDI). With every recurrence, the chance of a subsequent CDI episode increases. Early identification of patients at risk for rCDI might help doctors to guide treatment. The aim of this study was to externally validate published clinical prediction tools for rCDI.

**Methods:**

The validation cohort consisted of 129 patients, diagnosed with CDI between 2018 and 2020. rCDI risk scores were calculated for each individual patient in the validation cohort using the scoring tools described in the derivation studies. Per score value, we compared the average predicted risk of rCDI with the observed number of rCDI cases. Discrimination was assessed by calculating the area under the receiver operating characteristic curve (AUC).

**Results:**

Two prediction tools were selected for validation (Cobo 2018 and Larrainzar-Coghen 2016). The two derivation studies used different definitions for rCDI. Using Cobo’s definition, rCDI occurred in 34 patients (26%) of the validation cohort: using the definition of Larrainzar-Coghen, we observed 19 recurrences (15%). The performance of both prediction tools was poor when applied to our validation cohort. The estimated AUC was 0.43 [95% confidence interval (CI); 0.32–0.54] for Cobo’s tool and 0.42 (95% CI; 0.28–0.56) for Larrainzar-Coghen’s tool.

**Conclusion:**

Performance of both prediction tools was disappointing in the external validation cohort. Currently identified clinical risk factors may not be sufficient for accurate prediction of rCDI.

## Introduction

By 1978 *Clostridioides difficile* was considered to be a causative agent of antibiotic-associated pseudomembranous colitis.^1^ Nowadays we know this toxin-producing bacterium as the most common cause of healthcare-related diarrhea in the Western world.^2,3^ Primary *C. difficile* infection (CDI) is treated with antibiotics, either vancomycin or metronidazole.^4^ Despite adequate treatment, 15–25% of patients with CDI develop recurrent disease within 2 months.^5,6^ With every recurrence, the risk of a new CDI recurrence increases: the chance of developing a second recurrence is estimated at 45% and the risk of a third recurrence at 65%.^7^ The healthcare burden of recurrent CDI (rCDI) is substantial, since the 180-day mortality of patients with rCDI is 33% higher than that of patients with CDI without a recurrence.^8^ Multiple recurrences of CDI are treated with a tapered and/or pulsed regimen of vancomycin, fidaxomicin, or fecal microbiota transplantation (FMT).^4^ It is suggested that early treatment with fidaxomicin or FMT leads to lower recurrence rates.^9,10^ Early identification of patients at risk for rCDI is crucial as it permits specific preventive measures and treatment to be tailored for these patients.

Various studies have identified risk factors for rCDI. The most important contributors seem to be: older age, concomitant use of non-CDI antibiotics, antacids or immunosuppressive medication, severe underlying disease, and multiple or prolonged hospitalizations.^11–26^ Several models to predict rCDI have been developed.^13,16,20,27–32^ Unfortunately, none has gained clinical acceptance due to the limited number of patients on which they are based, insufficient performance, or lack of external validation. External validation of prediction tools for patients at risk of rCDI will give insight to the applicability of these tools in clinical practice and might contribute to better, personalized treatment for patients with CDI. Therefore, we aimed to search for prediction tools in the literature and to validate the most promising ones with a cohort of patients with CDI from six hospitals in The Netherlands.

## Methods

### Literature search

We performed a literature search in PubMed from database inception up to December 2019 (see Appendix). Only cohort studies with rCDI as an outcome measure that provided a practical scoring tool were selected. Prediction tools developed in a specific group of patients (e.g. trauma patients, ICU patients) or that used variables that were not available in our validation cohort were excluded. The study selection process was performed by two independent researchers and conflicts were handled by consensus.

### Validation cohort

For the validation cohort we used the already existing database of patients participating in an ongoing multicenter, prospective cohort study on the occurrence of rCDI, the PREDICD study (ZonMw project number 848016009). The aim of the PREDICD study is to develop a prediction model for rCDI based on a combination of clinical risk factors and fecal microbiota analysis. All adults (⩾18 years old) diagnosed with primary CDI that were hospitalized or visited the outpatient clinic of one of the participating centers between 1 March 2018 and 6 March 2020 were eligible for inclusion. Participating centers were: Amsterdam UMC location VUmc, OLVG, Spaarne Gasthuis, Haaglanden Medisch Centrum, Flevoziekenhuis, and Noordwest Ziekenhuisgroep. Primary CDI was defined as: (a) presence of diarrhea (defined as ⩾3 unformed stools within 24 h for a minimum of 2 consecutive days); (b) microbiologically confirmed CDI [using the diagnostic algorithm of the participating centre; enzyme immune assay (EIA) for glutamate dehydrogenase and/or free *C. difficile* toxin A and/or B, culture of toxigenic *C. difficile* and/or polymerase chain reaction (PCR) for detection of toxin A and/or B genes]; (c) treatment with metronidazole or vancomycin. Exclusion criteria were CDI in the preceding 3 months, microbiologically proven infectious colitis (other than CDI) in the last month, and ileostomy. This study was approved by the Medical Ethical Committee of Amsterdam UMC, location VUmc (approval number 2015.299). Written informed consent was obtained from all participants.

### Data collection

Data on patient characteristics and predicting variables used in the different prediction tools were collected prospectively by (telephone) interviews, and verified and completed with electronic patient healthcare records by a small group of trained researchers. Data were captured in Castor (Castor EDC, Amsterdam, the Netherlands), a secure and GCP-compliant (FDA 21 CFR Part 11, ICH E6 Good Clinical Practice, HIPAA and GDPR) clinical data management platform. Follow-up duration was 8 weeks, starting from the first day of treatment for primary CDI. Participants were contacted by telephone at scheduled time points (5, 10, 14, 28, and 56 days after treatment initiation). During these telephone consultations recovery and probable recurrence were evaluated. If patients were still hospitalized during the follow-up period, data were extracted from patient records. Participants were asked to contact the study coordinator if they developed diarrhea in between the scheduled time points.

### Statistical analysis

To evaluate the predictive value of the variables of the selected prediction tools in our validation cohort, we calculated the odds ratios (ORs) for these variables in the validation cohort with multivariable logistic regression analysis using the same multivariable models as used in the original studies. In addition, an rCDI risk score was calculated for each individual patient in the validation cohort using the scoring tool described in the derivation studies. Per score value, we compared the average predicted risk of rCDI with the observed number of rCDI cases. To quantify the ability of the prediction tools to differentiate between patients with and without rCDI (discrimination), we estimated the area under the receiver operating characteristic (ROC) curve (AUC), which ranges from 0.5 (no discrimination) to 1 (perfect discrimination). To quantify how close the calculated probabilities for rCDI were to the actual risk for rCDI, we plotted the observed number of rCDI cases *versus* the predicted number of rCDI cases (calibration plot).

## Results

### Selection description of prediction tools

From the literature search, 54 studies were identified by title and abstract screening. A total of 10 articles were selected for full-text review of which 2 were excluded since they did not include scoring tools and were therefore not suitable for validation analysis.^13,30^ We identified eight articles with a scoring tool for rCDI.^16,20,27–29,31–33^ Two articles reported the same prediction tool, therefore, only the original study was included.^16,33^ The Appendix shows the predictors identified in these seven studies. Older age was identified as a predictor for rCDI in 5/7 published prediction tools, while the other 26 predictors were used in only 1 or 2 prediction tools. Of the seven remaining studies, four were excluded because they used predictors that were not available in our cohort (i.e. Horn index score, use of antidiarrheals, fidaxomicin as therapy for CDI and abdominal distension).^16,20,29,32^ The prediction tool of Eyre *et al.* was excluded because it aimed to predict rCDI within 4 months of CDI diagnosis, instead of 2 months as used in our validation cohort.^28^ Eventually, two prediction tools were selected for validation analysis: the tool of Cobo *et al.* and that of Larrainzar-Coghen *et al.*^27,31^

### Selected prediction models

In the study of Cobo *et al.*, rCDI was defined as: (a) ⩾3 loose stools in 24 h or ileus or pseudomembranous colitis; (b) positive free toxin testing of stool (EIA) or nucleic acid amplification test for toxins (also called PCR) or culture of toxigenic *C. difficile* within 2 months after the completion of treatment for CDI. If a stool sample had not been sent to the laboratory for microbiological diagnostic confirmation, the reappearance of symptoms suggestive of rCDI that resolved with vancomycin or metronidazole treatment was also considered as rCDI. If a stool sample was negative for *C. difficile* despite response to treatment, the reappearance of diarrhea was not considered as rCDI.^27^ The prediction tool of Cobo *et al.* estimates the risk of rCDI by using the following predictors: age [<70 years (0 points), 70–79 years (1 point), and ⩾80 years (2 points)], positive EIA for free toxin in stool sample (1 point), episode of CDI in the previous year (2 points), and persistence of diarrhea (⩾3 unformed stools per 24 h) on the fifth day of treatment (2 points).^27^ Based on total points, Cobo *et al.* defined three risk categories: low risk (0–1 points), intermediate risk (2–3 points), and high risk (4–7 points). ^27^

Larrainzar-Coghen *et al.* defined rCDI as the presence of diarrhea (⩾3 loose stools per day for at least 2 consecutive days) combined with a positive EIA for toxins A and/or B within 8 weeks of the primary CDI, given that the symptoms of the first episode had resolved for at least 3 days.^31^ Larrainzar-Coghen *et al.* included four variables in their prediction tool: age (>65 years *versus* ⩽65 years), blood leukocyte count on the day of CDI diagnosis (⩽30× 10^9^/L *versus* >30 × 10^9^/L), enteral feeding 1 month preceding CDI diagnosis, and continuing proton pump inhibitor (PPI) treatment following CDI diagnosis.^31^ All variables were assigned 1 point for increased risk for rCDI implying a possible minimal score of 0 and a maximum of 4 points. Based on total points, two risk categories were defined: low risk (0–1 point) and high risk (2–4 points).^31^

### Missing data

The only missing data in the validation cohort were on blood leukocyte count (14 patients), 1 of the variables of Larrainzar-Coghen’s tool. Since we assumed that patients with severe leukocytosis would be seriously ill, and that their physicians would monitor their blood leukocyte count at least once every 3 days (we used a range of 3 days for measuring this value ‘at baseline’ in our validation cohort), we scored the 14 missing values as ⩽30 × 10^9^ leukocytes/L.

### Study and patient characteristics

Study and patient characteristics of the derivation studies and our validation cohort are shown in Table 1. The mean age of the participants in all three cohorts was comparable. The rates of rCDI in the validation cohort were similar to the rates of rCDI in the derivation cohorts. However, since Cobo *et al.* and Larrainzar-Coghen *et al.* used different definitions for rCDI, the rate of rCDI differed in the validation cohort, depending on the applied definition. According to Cobo’s definition, rCDI occurred in 26% of the patients in the validation cohort, whereas according to Larrainzar-Coghen’s definition only 15% of patients developed rCDI. The median time of recurrence was 19.5 days (range 11–50) after CDI treatment initiation when the rCDI definition of Cobo *et al.* was applied, and 18 days (range 11–44) after CDI treatment initiation when the rCDI definition of Larrainzar-Coghen was used.

**Table 1.** Study and patient characteristics of derivation and validation sets.

Variables	Derivation cohort Cobo *et al.*^27^ *N* (%)	Derivation cohort Larrainzar-Coghen *et al.*^31^ *N* (%)	Validation cohort PREDICD study *N* (%)
**Study setting**			
Inclusion period	2014–2015	2006–2013	2018–2020
Setting	Hospitalization + outpatient	Hospitalization	Hospitalization + outpatient
Location	14 Spanish hospitals	Spanish acute-care university hospital	6 Dutch hospitals
Number of patients	274	440	129
**Outcome**			
Recurrence of CDI *(Cobo definition)*	70 (25.6)		34 (26.4)
Recurrence of CDI *(Larrainzar-Coghen definition)*		61 (12.0)	19 (14.7)
**Patient characteristics**			
Age (years)	Mean 67.1 (SD 19.0)	Mean 62.3 (SD 18.5) (from *n* = 502*)	Mean 65.3 (SD 17.5)
Female sex	151 (55.1)	204 (46.4)	58 (45.0)
**CDI treatment**			
Metronidazole	162 (59.1)	434/502^*^ (86.5)	85 (65.9)
Vancomycin	76 (27.7)	26/502 (5.2)	44 (34.1)
Both	31 (11.7)	25/502 (5.0)	0
Other	0	9/502 (1.8)	0
Missing	5 (1.8)	8/502 (1.6)	0
**Predictors by Cobo *et al.*** ^27^			
Age (years)			
<70	128 (46.7)		64 (49.6)
70–79	51 (18.6)		39 (30.2)
⩾80	95 (34.7)		26 (20.2)
CDI episode in previous year			
Yes	29 (10.6)		2 (1.6)
No	245 (89.4)		127 (98.4)
Persistence of diarrhea on day 5			
Yes	114 (41.6)		57 (44.2)
No	159 (58.0)		72 (55.8)
Missing	1 (0.4)		0
Direct detection of toxin (EIA)			
Positive	152 (55.5)		82 (63.6)
Negative/not performed	122 (44.5)		47 (34.9)
**Predictors by Larrainzar-Coghen *et al.*** ^31^			
Age (years)			
⩽65		232 (52.7)	56 (43.4)
>65		208 (47.3)	73 (56.6)
Blood leukocyte count (× 10^9^/L)			
⩽30		413/431^$^ (95.8)	127 (98.4)
>30		18/431 (4.2)	2 (1.6)
Enteral feeding in last month			
No		399 (90.7)	106 (82.2)
Yes		41 (9.3)	23 (17.8)
Continuing PPI treatment			
No		135 (30.7)	51 (39.5)
Yes		305 (69.3)	78 (60.5)

* The complete cohort consisted of 502 patients; on these characteristics, only data of the full cohort were reported in the original study. However, patients who had a colectomy or died <30 days after inclusion were excluded from prediction tool development (*n* = 62).

$ Leukocyte count of nine patients was missing.

CDI, *Clostridioides difficile* infection; EIA, enzyme immune assay; PPI, proton pump inhibitor; SD, standard deviation.

With respect to the risk factors used in the prediction tools, in the study of Cobo *et al.*^27^ 11% of patients had a CDI episode in the previous year, in contrast to only 2% of patients in the validation cohort. An important note here is that in the validation cohort patients with CDI in the previous 3 months were excluded, since we aimed to include in the rCDI follow up only patients with a primary CDI.

### Prediction tools performance

#### Prediction tool of Cobo *et al.*^27^

**Figure 1.**
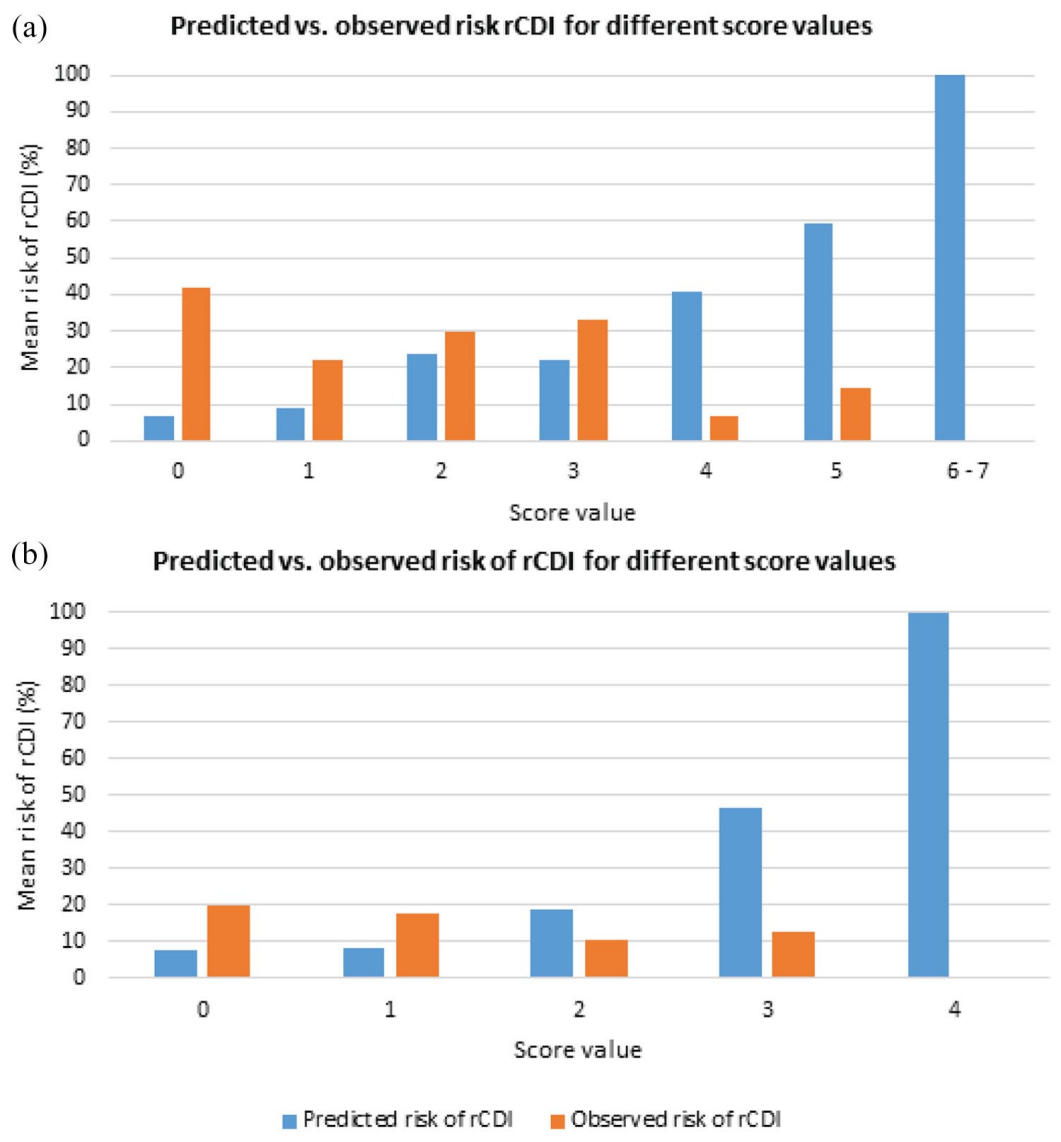
Validation of the prediction tools of Cobo *et al.*^27^ (a) and Larrainzar-Coghen *et al.*^31^ (b) by comparing the average predicted *versus* observed risk of rCDI per risk score value. rCDI, recurrent *Clostridioides difficile* infection.

We calculated the ORs of Cobo’s predictors for rCDI in the validation cohort by using multivariable logistic regression analysis (Table 2). None of Cobo’s predictors was associated with rCDI in our validation cohort. Thereafter, we calculated rCDI risk scores for each individual patient in the validation cohort. Per score value, we compared the average predicted risk of rCDI with the observed number of rCDI cases (Figure 1(a) and Appendix). In the validation cohort, a higher score corresponded to a lower risk of rCDI; the highest risk was observed for the patients with a score of 0 (predicted as *low risk*). The estimated AUC of 0.43 [95% confidence interval (CI); 0.32–0.54] confirmed the poor discrimination between patients with and without rCDI. This poor discriminative ability differs substantially from the performance observed in Cobo’s study where in the derivation cohort an AUC of 0.72 (95% CI; 0.65–0.79) was estimated, with an AUC of 0.75 (95% CI; 0.67–0.83) in their internal validation cohort.

**Table 2.** Comparison of odds ratios of the predictors between the derivation cohorts and the validation cohort.

	Scores derivation study	Validation set, *N* (%)		OR of predictors in derivation cohort (95% CI)	OR of predictors in validation cohort (95% CI)
		Recurrence (definition Cobo *et al.*^27^)			
		No 95 (73.6)	Yes 34 (26.4)		
**Predictors Cobo *et al.*** ^27^					
Age (years)					
<70	0	44 (46.3)	20 (58.8)	1 (Reference)	1 (Reference)
70–79	1	30 (31.6)	9 (26.5)	1.63 (1.42–3.78)	0.49 (0.22–1.12)
⩾80	2	21 (22.1)	5 (14.7)	3.22 (1.65–6.23)	0.39 (0.14–1.10)
CDI episode in previous year					
No	0	93 (97.9)	2 (5.9)	1 (Reference)	1 (Reference)
Yes	2	2 (2.1)	0 (0)	3.67 (1.57–8.50)	0.00 (0.00–)
Persistence of diarrhea on day 5					
No	0	52 (54.7)	20 (58.8)	1 (Reference)	1 (Reference)
Yes	2	43 (45.3)	14 (41.2)	3.25 (1.79–5.93)	0.71 (0.34–1.46)
Direct detection of toxin (EIA)					
Negative/not performed	0	34 (35.8)	13 (38.2)	1 (Reference)	1 (Reference)
Positive	1	61 (64.2)	21 (61.8)	1.92 (1.09–4.06)	0.56 (0.31–1.04)
		Recurrence (definition Larrainzar-Coghen *et al.*^31^)		OR of predictors in derivation cohort (95% CI)	OR of predictors in validation cohort (95 % CI)
		No 110 (85.3)	Yes 19 (14.7)		
**Predictors Larrainzar-Coghen *et al.*** ^31^					
Age (years)					
⩽65	0	45 (40.9)	11 (57.9)	1 (Reference)	1 (Reference)
>65	1	65 (59.1)	8 (42.1)	2.04 (1.14–3.68)	0.22 (0.09–0.52)
Blood leukocyte count (× 10^9^/L)					
⩽30	0	109 (99.1)	18 (94.7)	1 (Reference)	1 (Reference)
>30	1	1 (0.9)	1 (5.3)	2.85 (0.97–8.38)	7.64 (0.39–148.95)
Enteral feeding in last month					
No	0	90 (81.8)	16 (84.2)	1 (Reference)	1 (Reference)
Yes	1	20 (18.2)	3 (15.8)	3.62 (1.66–7.87)	0.48 (0.13–1.83)
Continuing PPI treatment					
No	0	42 (38.2)	9 (47.4)	1 (Reference)	1 (Reference)
Yes	1	68 (61.8)	10 (52.6)	1.89 (0.93–3.87)	0.36 (0.16–0.82)

CDI, *Clostridioides difficile* infection; CI, confidence interval; EIA, enzyme immune assay; OR, odds ratio; PPI, proton pump inhibitor.

#### Prediction tool of Larrainzar-Coghen *et al.*^31^

The ORs of the predictors of Larrainzar-Coghen *et al.*^31^ for rCDI in the validation cohort are shown in Table 2. Enteral feeding was not significantly associated with rCDI in our validation cohort. Only two patients in our cohort had a blood leukocyte count of >30 × 10^9^/L. Age >65 years and continuing PPI treatment were associated with the absence of rCDI with ORs of 0.22 (95% CI; 0.09–0.52) and 0.36 (95% CI; 0.16–0.82), respectively. Also for this prediction tool, the average predicted risk of rCDI per score value did not correspond well with the observed number of rCDI cases (Figure 1(b) and Appendix). In line, discrimination was poor with an estimated AUC of 0.42 (95% CI; 0.28–0.56). This finding is in contrast with the performance of the prediction tool in the original derivation cohort in which an AUC of 0.67 (95% CI; 0.59–0.75) was estimated. Since the prediction tool of Larrainzar-Coghen *et al.*^31^ was developed in a cohort of hospitalized patients only, we also performed a validation restricted to the hospitalized patients of our validation cohort (*n* = 113). This did not influence the ORs of the predictors of the Larrainzar-Coghen *et al.*^31^ model in our cohort, neither did it substantially influence the discriminative performance of the model (AUC = 0.47: 95% CI; 0.31–0.62).

The calibration curves confirm the poor performance of both tools for predicting rCDI in the validation cohort (see Appendix).

## Discussion

This study aimed to externally validate two existing prediction tools for rCDI. The tools of Cobo *et al.*^27^ and Larrainzar-Coghen *et al.*^31^ performed poorly in our validation cohort with estimated AUCs of 0.43 (95% CI; 0.32–0.54) and 0.42 (95% CI; 0.28–0.56), respectively. Remarkably, ROC and calibration plots of both prediction models showed a negative correlation: lower predicted probabilities for rCDI correlated with higher observed risks for rCDI, whereas higher predicted probabilities correlated with lower actual risks for rCDI. This suggests that, despite the similarities in study settings, these prediction tools are not sufficient for accurate prediction of rCDI in the general population.

The drawback of most prediction tools for rCDI is the lack of external validation.^20,28,29,31,32^ To the best of our knowledge, our study is the first in which prediction tools for rCDI were validated in a setting completely independent from the setting in which the tools were developed. This might be a reason for the poor performance of these tools in our population. In only 2/7 published prediction tools for rCDI, namely those of Cobo *et al.*^27^ and Hu *et al.*,^16^ was an ‘external’ validation performed. Both tools discriminated well between patients with and without rCDI in their own validation cohorts with AUCs of 0.75 (95% CI; 0.67–0.83) and 0.80 (95% CI; 0.67–0.92). However, in both studies the validation cohorts were highly similar to the derivation cohorts, because they were largely chosen from the same source population. To determine the true robustness of a prediction model, derivation and validation cohorts should be derived from different populations.

That different study settings lead to different rCDI predictors is nicely illustrated in the Appendix: most predictors are ‘unique’ and included in only one or two prediction tools. This can be partially explained by the fact that not all studies collected the same variables. However, data on the ‘usual suspects’, such as antibiotic and PPI use, signs and symptoms of severe CDI, and immune status, were collected in the majority of these studies but generally not identified as predictors in multivariable analysis. This high variation in rCDI predictors might reflect the heterogeneity of the patient population and study designs, and could be an explanation for the low generalizability of these tools in other populations.

To explain the poor performance of the prediction tools in our validation cohort, we compared the study and patient characteristics of both derivation cohorts and the validation cohort (Table 1). Data from all three studies were collected prospectively in European hospitals between 2006 and 2022. The cohort of Larrainzar-Coghen *et al.*^31^ consisted exclusively of hospitalized patients and was carried out in a university hospital, while the other cohorts consisted of both inpatients and outpatients recruited from a combination of university and general hospitals. Furthermore, the two derivation studies used a different definition for rCDI (see Results section). However, despite these different definitions, recurrence rates in the validation cohort were highly similar to those in the derivation cohort according to the definition applied: Larrainzar-Coghen *et al.*’s cohort: 12% *versus* validation cohort: 15%, and Cobo *et al.*’s cohort: 26% *versus* validation cohort: 26%. Concerning the predictors, the cohort of Cobo *et al.* comprised more patients of ⩾80 years of age (35%), when compared with the validation cohort (20%). This might be explained by the fact that in the PREDICD study patients were asked to collect an extra stool sample for microbiota analysis; some frail older patients therefore refused study participation. This might have resulted in the inclusion of solely ‘healthy’ patients aged 80+ years with lower risk of recurrent disease. Besides older age, Cobo *et al.* identified ‘CDI in the last year’ as a predictor of rCDI. In the PREDICD study we excluded patients with CDI in the preceding 3 months. This might be the reason that Cobo’s cohort comprised more patients with a CDI episode in the last year (11%) than in the validation cohort (2%), and is a limitation of our study. Since rCDI is a major risk factor for a subsequent recurrence, this might explain why ‘CDI in the last year’ was identified as a risk factor in Cobo’s cohort but not in our population.

In the cohort of Larrainzar-Coghen *et al.*,^31^ older age and PPI continuation after CDI diagnosis were risk factors for rCDI. In our cohort these variables were inversely associated with rCDI. This is remarkable since older age is identified as risk factors for rCDI in many previous studies.^16,28,29^ The literature on the association between PPI use and rCDI is less consistent.^12,23,32,34,35^ Considering age, Larrainzar-Coghen *et al.*^31^ dichotomized the variable age into ⩽65 years and >65 years of age. We scrutinized the continuous values and observed that this cut-off was quite arbitrary in our cohort: for example, when the cut-off value for age would have been >60 years old instead of >65 years, the patients with rCDI categorized as ‘older’ would shift from 42% to 68% and ‘older’ age would have been a (positive) predictor for rCDI. Therefore, we suggest the use of continuous values or multiple age categories in future prediction tools for more accurate and individualized prediction of rCDI risk.

A difference between our study and that of Larrainzar-Coghen *et al.*^31^ is that we also included patients that visited the outpatient clinic (*n* = 16). Despite our expectations, rCDI occurred more frequently in outpatients (25%) than in hospitalized patients (14%). This could be a result of our active, prospective approach: patients with mild, possibly self-limiting complaints might have consulted a doctor more frequently due to our telephone consultations than they would have in a normal situation. Another explanation might be that spores are difficult to eliminate and re-exposure to spores in the home environment may be a source for relapse. However, when we performed a validation analysis restricted to the hospitalized patients of the validation cohort (*n* = 113), this did not increase the performance of the prediction tool.

Besides the clinical features used in prediction tools for rCDI, other variables may be predictive for rCDI. The development of antitoxin antibodies seems to be an important factor for disease resolution and the prevention of rCDI.^36,37^ Furthermore, it is known that toxin production, sporulation, persistence in the host and spore germination are elevated in several hypervirulent strains such as 027/NAP1 and 078 and may influence the risk of rCDI.^38–41^ Since changes in gut microbiota composition play an important role in the pathogenesis of (recurrent) CDI, Khanna *et al.* developed a microbiota-based risk score for rCDI that showed promising results.^42^ Because many clinical factors (such as age and medication use) have an effect on the diversity of the gut microbiota and therefore on the risk of rCDI, incorporation of microbiota-related risk factors in prediction tools could lead to a more direct and accurate prediction of rCDI. We hope to confirm this hypothesis in the near future with the results of the PREDICD study. Another interesting predictor might be the virome, however, this is not yet generally considered in microbiota studies.

One of the strengths of this study is the prospective data collection by both telephone interviews and electronic health records, resulting in a few missing data. In addition, all patients in our cohort had symptomatic CDI, therefore, the risk of including patients with *C. difficile* colonization instead of infection was low. A limitation of our study was the relatively small sample size of 129 patients and the lack of a sample size calculation due to the use of a ‘convenience sample’ consisting of patients in the PREDICD study cohort. Another limitation is that we were able to validate only two of the seven prediction tools found *via* the literature search, mainly because they used predictors that were nonquantitative and/or variables that we did not collect for the patients in our cohort.

In conclusion, our results show poor performance of two practical prediction tools for rCDI. Accurately predicting recurrent disease remains a challenge. Possibly, prediction models with more parameters, such as microbiota composition at time of CDI diagnosis, are needed for better prediction of rCDI.

## Figures and Tables

**Figure:**
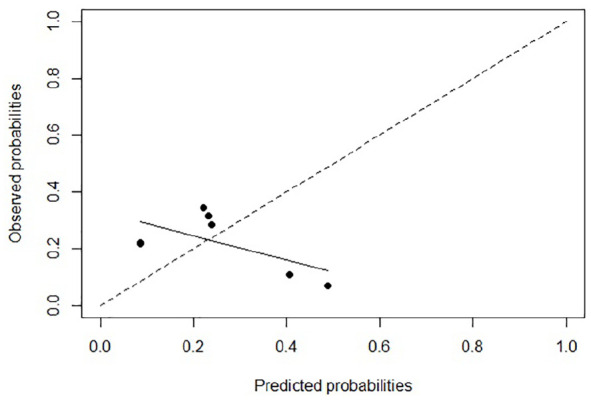
Cobo *et al.*^27^

**Figure:**
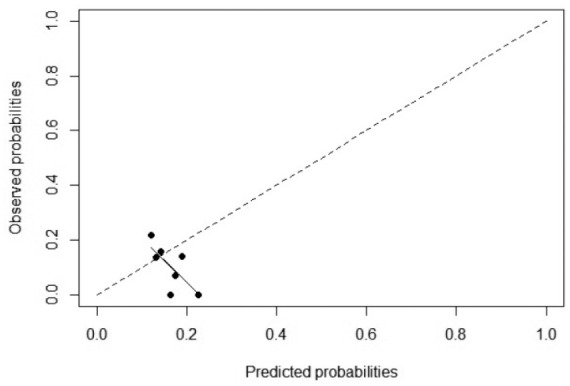
Larrainzar-Coghen *et al.*^31^

## Appendix

### Literature search

(“Decision Support Techniques”[MeSH] OR “Decision Support”[tiab] OR “Decision Aids”[tiab] OR “Decision Aid”[tiab] OR “Decision Analyses”[tiab] OR “Decision Analyse”[tiab] OR “Decision Model”[tiab] OR “Decision Models”[tiab] OR “Decision Modelling”[tiab] OR “Prediction Rule”[tiab] OR “Prediction Rules”[tiab] OR “prediction model”[tiab] OR “prediction models”[tiab] OR “prediction modeling”[tiab] OR “prediction tool”[tiab] OR “prediction tools”[tiab] OR “risk score”[tiab] OR “risk scores”[tiab] OR “risk scale”[tiab] OR “risk scales”[tiab] OR “risk index”[tiab] OR “scoring system”[tiab] OR “prediction score”[tiab] OR “decision score”[tiab] OR “scoring tool”[tiab] OR “scoring index”[tiab]) AND ((rCDI[tiab] OR RCDI[tiab] OR “R-CDI”[tiab]) OR ((Recurrence[MeSH] OR Recurr*[tiab] OR Recrudescence*[tiab] OR Repeat*[tiab] OR Relaps*[tiab] OR Repetit*[tiab] OR reappear*[tiab] OR Period*[tiab] OR Return*[tiab]) AND (“Clostridium difficile”[MeSH] OR “Clostridium difficile”[tiab] OR “Peptoclostridium difficile”[tiab] OR “C. diff”[tiab] OR “C. difficile”[tiab] OR CDI[tiab] OR C-diff[tiab] OR “Clostridioides difficile”[tiab] OR Pseudomembran*[tiab] OR “Enterocolitis, Pseudomembranous”[MeSH] OR PMC[tiab])))

**Table:** Predictors identified in seven published prediction tools for rCDI

Studies→ ↓ Used predictors	Cobo *et al.*^27^	Reveles *et al.*^43^	Viswesh *et al.*^32^	Larrainzar-Coghen *et al.*^31^	D’Agostino *et al.*^29^	Eyre *et al.*^28^	Hu *et al.*^16^	Number of studies using the predictor
Age	X			X	X	X	X	5/7
Prior CDI	X				X			2/7
Persistence of diarrhea	X							1/7
Free toxin in stool (+EIA)	X							1/7
Blood leukocyte count			X	X				2/7
Enteral feeding				X				1/7
Use of PPIs/antacid		X		X				2/7
Prior use of cephalosporins		X						1/7
Prior use of antidiarrheals		X						1/7
Nonsevere CDI		X						1/7
Status ‘community-onset’		X						1/7
Emergency admission						X		1/7
Previous MRSA+						X		1/7
Previous dialysis/chemotherapy						X		1/7
Number of unformed stools/day					X	X		2/7
Presence of CDI at admission			X			X		2/7
C-reactive protein						X		1/7
(Duration of) Past admission						X		1/7
Elective admission/community sample AND previous MRSA isolated (protective)						X		1/7
Status ‘indeterminate disease’						X		1/7
Body temperature			X					1/7
Status ‘nosocomial CDI’			X					1/7
Abdominal distension			X					1/7
Blood creatinine level					X			1/7
Choice of treatment vancomycin/fidaxomicin					X			1/7
Horn’s index							X	1/7
Continued use of antibiotics							X	1/7

CDI, *Clostridioides difficile* infection; EIA, enzyme immune assay; MRSA, methicillin-resistant *Staphylococcus aureus*; PPI, proton pump inhibitor; rCDI, recurrent *Clostridioides difficile* infection; X, variable included in prediction model.

**Table:** Predicted *versus* observed risk of rCDI by risk score Cobo *et al*.^27^

Score	Cases, *n*	Observed rCDI, *n* (%)	Average predicted rCDI, *n* (%)
0	12	5 (41.7)	1 (6.7)
1	32	7 (21.9)	3 (8.7)
2	27	8 (29.6)	6 (23.8)
3	36	12 (33.3)	8 (22.1)
4	15	1 (6.7)	6 (40.6)
5	7	1 (14.3)	4 (59.4)
6–7	0	0	0 (100)
**Total**	129	34 (26%)	28 (22%)

rCDI, recurrent *Clostridioides difficile* infection.

**Table:** Larrainzar-Coghen *et al.*^31^

Score	Cases (%)	Observed rCDI, *n* (%)	Average predicted rCDI, *n* (%)
0	25 (19.1)	5 (20.0)	2 (7.7)
1	40 (31.0)	7 (17.5)	3 (8.5)
2	56 (43.3)	6 (10.7)	10 (18.6)
3	8 (6.2)	1 (12.5)	4 (46.7)
4	0	0	0 (100)
**Total**	129	19 (14.7%)	19 (14.7%)

rCDI, recurrent *Clostridioides difficile* infection.

### Calibration plots

Calibration of the prediction tool of Cobo *et al.*^27^ and Larrainzar-Coghen *et al.*^31^ using the original scores. Dotted lines: ‘ideal’ calibration curves; solid lines: calibration curves of the prediction tools.
